# Reply to Vale et al. Comment on “Zhong et al. Sugar-Sweetened and Diet Beverage Consumption in Philadelphia One Year after the Beverage Tax. *Int. J. Environ. Res. Public Health* 2020, *17*, 1336”

**DOI:** 10.3390/ijerph182010930

**Published:** 2021-10-18

**Authors:** Yichen Zhong, Amy H. Auchincloss, Brian K. Lee, Ryan M. McKenna, Brent A. Langellier

**Affiliations:** 1 Department of Epidemiology and Biostatistics, Dornsife School of Public Health, Drexel University, Philadelphia, PA 19104, USA; yz624@drexel.edu (Y.Z.); bkl29@drexel.edu (B.K.L.); 2 Department of Health Management and Policy, Dornsife School of Public Health, Drexel University, Philadelphia, PA 19104, USA; rmmckenna@gmail.com (R.M.M.); bal95@drexel.edu (B.A.L.)

Thank you for the opportunity to respond to the recent letter to the editor regarding our paper “Sugar-Sweetened and Diet Beverage Consumption in Philadelphia One Year after the Beverage Tax”. 

The first concern raised in the letter was “lack of a paragraph on the sample calculation”. The methods section of our paper notes that our study is based on a random-digit-dialing phone survey (and a reference pointed to more detail in a previously published paper [1]). The criteria for selecting the 515 participants as the final analytic sample were described in Figure S1. The loss of follow-up was expected to be high, and the attrition observed in our study was similar to other random-digit-dialing phone surveys [2,3]. We described the power calculation in the methods section and provided more details in the supplement (Figure S2). To summarize, the sample size of our study was powered to detect a quite small difference, three times per week, which was similar to the effect size reported in prior studies [4,5].

The second concern was that “quasi-experimental study protocols should be registered”. We do not disagree with the importance of randomized trial registration, however, for non-randomized designs registration is not required nor is it customary [6]. 

Our study—using a population sample—found no substantial change in beverage consumption in Philadelphia one year after the tax. We do not agree with the letter that our finding diverged from the weight of the evidence reported to date. There is currently no consensus on the long-term impact of tax on beverage consumption. Based on previous literature, the impact of sugary beverage taxes seems to vary based on baseline levels of consumption, population characteristics, as well as the magnitude of the tax and uniformity of implementation. This heterogeneity has been broadly documented and recognized in recent review articles [7]. Studies conducted in samples with low baseline consumption tend to report no substantial mid- to long-term change after tax [4,8,9,10].

Further, as mentioned in our discussion section, when taxes are not uniformly implemented, tax avoidance—such as cross-border shopping—can attenuate the tax’s impact. Cross-border shopping could be an important reason for the attenuated impact of the tax observed in multiple studies [11,12]. Our study also observed potential evidence for increased cross-border shopping.

Industry groups opposed to the tax should not be encouraged by our study and tax advocates should not be discouraged. We noted in our paper—and re-state for emphasis here—that observing no substantial change in a sample with low baseline consumption like ours does not mean that the tax has no impact in sub-populations. Indeed, it was reported that the Philadelphia sugary beverage tax decreased sugary beverage consumption in very low income populations that had children living at home [4].

Our methods were fully disclosed, and the strengths and limitations were discussed extensively in the paper. There are very few population-based studies of consumer responses to sugary beverage taxes. We believe that our study is an important addition to the literature by providing rigorous and transparent research on the impact of the beverage tax on the general population with lower baseline consumption. We encourage research replication in other contexts in population-based samples, as well as in higher-risk samples.
